# Rapid genetic and phenotypic changes in *Pseudomonas aeruginosa* clinical strains during ventilator-associated pneumonia

**DOI:** 10.1038/s41598-019-41201-5

**Published:** 2019-03-18

**Authors:** Elise Persyn, Mohamed Sassi, Marc Aubry, Martin Broly, Sandie Delanou, Karim Asehnoune, Nathalie Caroff, Lise Crémet

**Affiliations:** 1 EA3826 Université de Nantes, IRS2 Nantes Biotech, Nantes Cedex 1, F-44100 France; 2 CHU Nantes, 9 quai Moncousu, Nantes Cedex 1, F-44093 France; 3 Inserm U835, Université de Rennes, Rennes, F-35000 France; 4 Université de Rennes, CNRS, IGDR [(Institut de génétique et développement de Rennes)] - UMR 6290, F-35000 Rennes, France; 5 Université de Rennes, Plateforme GEH, CNRS, Inserm, BIOSIT - UMS 3480, US_S 018, F-35000 Rennes, France

## Abstract

Treatment with antibiotics leads to the selection of isolates with increased resistance. We investigated if evolution towards resistance was associated with virulence changes, in the context of *P*. *aeruginosa* ventilator-associated pneumonia (VAP). Four patients were selected because they had multiple VAP episodes during short periods (12 days to 5 weeks), with emergence of resistance. We performed whole-genome sequencing of 12 *P*. *aeruginosa* from bronchoalveolar lavages or blood culture (3 isolates per patient). Production of *quorum sensing*-dependent virulence factors, serum resistance, cytotoxicity against A549 cells, biofilm production, and twitching motility were studied. Each patient was infected with a unique strain. For all patients, resistance development was explained by genetic events in *ampD*, *mexR* or *oprD*. Additional variations were detected in virulence- and/or fitness-associated genes (*algB*, *gacA*, *groEL*, *lasR*, *mpl*, *pilE*, *pilM*, *rhlR*) depending on the strain. We noticed a convergence towards *quorum sensing* deficiency, correlated with a decrease of pyocyanin and protease production, survival in serum, twitching motility and cytotoxicity. In one patient, changes in *pilM* and *pilE* were related to enhanced twitching. We show that the emergence of resistance in *P*. *aeruginosa* is associated with virulence modification, even in acute infections. The consequences of this short-term pathoadaptation need to be explored.

## Introduction

*Pseudomonas aeruginosa* is an opportunistic pathogen and one of the main agents of ventilator-associated pneumonia (VAP)^1,2^. VAP causes significant morbidity with longer intensive care unit stay and additional hospital costs of at least US $10,019^3^.

*P*. *aeruginosa* has a broad arsenal of virulence factors. Their production is finely regulated, in particular by the *quorum sensing*. This network is composed of four interconnected systems, each consisting of a regulatory protein (transcription factor) and an autoinducing enzyme: LasR / LasI for the *las* system and RhlR / RhlI for the *rhl* system. *Quorum sensing* regulates 10% of *P*. *aeruginosa* genome based on bacterial density and probably on environmental stress cues^4^.

Antibiotic resistance is another key factor in the evolution of *P*. *aeruginosa* infections. In recent years, multidrug-resistant *P*. *aeruginosa* clones have caused outbreaks of healthcare-associated infections worldwide^5^. This leads to wonder about the intrinsic virulence of these multidrug-resistant strains^6^.

The relationship between virulence and resistance in *P*. *aeruginosa* is still poorly understood. It is generally thought that resistance implies an energy cost and impairs fitness, negatively impacting virulence. Three studies in rabbit or mouse models showed that multidrug-resistant *P*. *aeruginosa* isolates were less virulent, but these studies included a small number of genetically unrelated isolates^7–9^. The *in vitro* study of Mulet *et al*. performed on 40 clonally diverse *P*. *aeruginosa* showed that multidrug-resistant isolates had reduced motility and fitness^5^. On the other hand, Skurnik *et al*. showed that carbapenem-resistant *oprD* mutants had a greater ability to colonise the mouse gut and to disseminate to the spleen than non-mutated isogenic isolates^10^. Carbapenem-resistant isolates displayed increased cytotoxicity against murine macrophages and were more resistant to human serum in this study. In addition, *P*. *aeruginosa* protein AmpR positively regulates the expression of resistance and virulence genes^11^. Some specific resistance mechanisms, like loss of OprD or AmpC overexpression, could therefore be associated with modification of virulence.

Pathoadaptation phenomenon has been widely described in hypermutable *P*. *aeruginosa* strains, during chronic pulmonary infections in cystic fibrosis patients, with switch to mucoidity, antibiotic resistance and reduced production of virulence factors^12^, but has been poorly studied in the context of acute infections like VAP. Only recently, Wang *et al*. investigated the adaptive evolution of a single *P*. *aeruginosa* strain responsible for a nosocomial outbreak of VAP infections in China. They found convergent genomic events leading to attenuated virulence^13^.

Thus, in order to see if acquisition of resistance mechanisms could be associated with changes in strain virulence, we analysed the genetic and phenotypic characteristics of 4 *P*. *aeruginosa* strains that developed antibiotic resistance during acute VAP infections in 4 critically ill patients.

## Materials and Methods

### Bacterial isolates

The 12 *P*. *aeruginosa* isolates studied here were collected at Nantes Universitary Hospital in 4 VAP patients hospitalised in intensive care units. For each patient, 3 different isolates were recovered over periods of 12 days to 5 weeks. Eleven isolates were collected from bronchoalveolar lavages (BAL) and one was recovered from blood culture (Table 1). In 2 cases, 2 isolates with different colony morphologies were collected from the same BAL. The clonal relatedness of isolates was investigated by MLST (MultiLocus sequence typing) using the online *P*. *aeruginosa* MLST database (www.pubmlst.org). This study was performed on strains isolated from samples sent to the bacteriology lab for diagnosis, without any additional sampling. In Nantes Universitary Hospital, an informed consent is asked for use of data for research purpose on all patients.

**Table 1:** List of studied *P*. *aeruginosa* isolates with antibiotic susceptibility profiles, antibiotics received by patients, genomic variations found between the earliest isolate and late isolates of each patient, and clinical evolution.

Isolate	Clinical specimen	Days of mechanical ventilation	Sequence Type	MICs (mg/L)^*a*^						Antibiotic treatment (initiation day)^*a*^	Genomic variation^*b*^	Coding region change	Amino acid change^*c*^	Clinical evolution
				TIC	TCC	PIP	CAZ	IPM	CIP					
***Patient 1*** **(** ***strain PA-VAP-1*** **)**														
1A	BAL	3 days	1027	16	16	≤4	2	2	≤0.25	CAZ (day 6)	—			death in intensive care unit
1B	BAL	14 days	1027	16	16	≤4	≤1	2	≤0.25		—			
1C	BAL	14 days	1027	>64	>64	>64	32	2	≤0.25	IPM/AN (day 17)	SNP	*ampD*: 358 G > T	AmpD: Glu120*	
											SNP	*gacA*: 601 T > G	GacA: Ser201Ala	
***Patient 2*** **(** ***strain PA-VAP-2*** **)**														
2A	BAL	4 days	2960	32	32	8	4	0.5	≤0.25	CAZ/CS (day 5)	—			death in intensive care unit
2B	BAL	27 days	2960	64	32	16	4	≥16	≤0.25		INS	*oprD*: 672_673insC	OprD: Tyr225fs	
											SNP	*groEL*: 560 C > T	GroEL: Pro187Leu	
2C	BAL	27 days	2960	32	32	16	4	0.5	≤0.25	FEP/CS (day 30)	SNP	*groEL*: 560 C > T	GroEL: Pro187Leu	
											SNP	*pilM*: 808 C > T	PilM: Gln270*	
											INS	*pilE*: 208_209insTCGG	PilE: Thr70fs	
***Patient 3*** **(** ***strain PA-VAP-3*** **)**														
3A	BAL	1 day	253	32	32	16	4	1	2	IPM/TN (day 1)	—			discharge from intensive care unit
3B	BAL	11 days	253	32	16	8	4	1	2	IPM (day 11); CAZ/CS (day 13)	DEL	*lasR*: 138_148delCTACGAGAACG	LasR: Tyr47fs	
											INS	*algB*: 853_854insCCGCGACCAA	AlgB: Trp288fs	
3C	BAL	35 days	253	32	32	8	4	≥16	2	FEP (day 37)	DEL	*lasR*: 138_148delCTACGAGAACG	LasR: Tyr47fs	
											INS	*algB*: 853_854insCCGCGACCAA	AlgB: Trp288fs	
											DEL	*oprD*: 16_132del117-bp	OprD: loss of 39 AA	
											SNP	*mpl*: 314 T > G	Mpl: Val105Gly	
***Patient 4*** **(** ***strain PA-VAP-4*** **)**														
4A	BAL	21 days	2042	32	32	16	2	1	≤0.25	FEP/LVX (day 22)	—			discharge from intensive care unit
4B	BAL	46 days	2042	≥128	≥128	64	8	1	1	IPM (day 46)	SNP	*lasR*: 676 G >A	LasR: Val226Ile	
											DEL	*rhlA*, *rhlB*, and *rhlR*: 175_1 del 2186-bp	loss of RhlR & RhlB RhlA: Phe59fs	
											INS	*mexR*: 80_81insT	MexR: Glu27fs	
4C	blood culture	48 days	2042	32	16	8	2	1	≤0.25		—			

^*a*^AN, amikacin; CAZ, ceftazidime; CIP, ciprofloxacin; CS, colistin; FEP, cefepime; IPM, imipenem; LVX, levofloxacin; PIP, piperacillin; TCC, ticarcillin-clavulanate; TIC, ticarcillin; TN, tobramycin.

^*b*^DEL, deletion; INS, insertion; SNP, Single Nucleotide Polymorphism.

^*c*,*^STOP codon; AA, amino acids; fs, frameshift mutation.

### Sequencing, assembly and annotation

Bacterial DNA was extracted with iPrep™ PureLink® Virus Kit (Thermo Fisher Scientific) from a single colony of each isolate. DNA concentration was measured by fluorometric method on Quantus™ (Promega®). One μg of each DNA extract was fragmented by sonication on Bioruptor® (Diagenode). The libraries were prepared using TruSeq® DNA PCR-Free Library Prep kit (Illumina®) and sequenced on MiSeq® (Illumina®) with MiSeq Reagent Kit v2 300 cycles (paired-end sequencing 2 × 150 cycles).

All sequencing data used in this study are available on NCBI BioProject n° PRJNA438112. The Illumina reads were trimmed using Trimmomatic^14^, quality filtered with the FASTX-Toolkit (http://hannonlab.cshl.edu/fastx_toolkit/) and assembled using SPAdes^15^. SIS software^16^ and GapFiller version 1.10^17^ were used to improve the initial set of contigs before sending for annotation using the NCBI Prokaryotic Genome Annotation Pipeline (PGAP)^18^.

### Comparative and phylogenetic analysis

To investigate the epidemiological linkage of our 12 isolates and commonly used strains (e.g. PAO1, PA14, ATCC_27853, NCTC_10332), environmental strains (e.g. B10W, YL84) or clinical strains (e.g. PA_D1 sampled from VAP patients; PA1 from a patient with respiratory tract infection; RP73, DK2 and FRD1 from cystic fibrosis patients), 43 complete genome sequences were retrieved from NCBI (http://www.ncbi.nlm.nih.gov/genome). Core-genome alignment was carried out using PARSNP v. 1.2^19^ and PAO1 genome as reference. Recombinant sites were identified using PhiPack^20^ and single-nucleotide polymorphisms (SNPs) located in those regions were filtered yielding 172,245 single-nucleotide variants. Phylogenetic analysis was performed considering the 172,245 polymorphic sites of the 55 genomes. A transversion substitution model was selected on the basis of the Akaike’s information criterion with jModelTest2^21^. Maximum likelihood phylogeny was constructed using PhyML^22^ and visualized using Figtree (http://tree.bio.ed.ac.uk/software/figtree/). The 12 *P*. *aeruginosa* genomes of this study were also submitted to CSIphylogeny (https://cge.cbs.dtu.dk/services/CSIPhylogeny/) for pan genomic analysis. Antibiotic resistance genes were predicted from PAO1 genome and the assembled genomes using the ResFinder 3.0 server [https://cge.cbs.dtu.dk/services/ResFinder/]. Comparison of PAO1 genome against our 4 *P*. *aeruginosa* strains genomes was done by BLAST search using BLAST Ring Image Generator 0.95^23^. Genomes were annotated using Prokka v1.12 software^24^ and proteomes were used for pan-genomic analysis with OrthoMCL software^25^. Homologous sequences were selected using the all-against-all BlastP algorithm with an E value of <10^−5^ and 60% nucleotide identity. Functional annotation was performed using COG database (http://weizhongli-lab.org/metagenomic-analysis/server/cog/).

### Detection of nucleotide differences

Nucleotide differences were detected and evaluated using CLC GENOMIC WORKBENCH. For each patient, the assembled early isolate genome was annotated and used as reference genome for the other 2 isolates. The paired-end reads in FASTQ format were mapped to the early isolate genome and further compared with each other to generate lists of SNPs and short indels. A frequency cut-off of more than 80% was set to minimise false SNPs due to sequencing error. For confirmation of nucleotide differences, the 3 BAM files from each patient were visualised and compared using the Integrative Genomics Viewer (IGV) version 2.3 (Broad Institute, Cambridge, MA), and questionable regions were analysed by PCR/Sanger sequencing. In order to compare with the reference genome, all paired-end reads were mapped to the annotated genome PAO1.

### Antimicrobial susceptibility testing

Antibiotic susceptibility was determined by VITEK^®^ 2 XL (bioMérieux, Marcy l’Etoile, France).

### Resistance to human serum

Resistance to pooled human serum was assessed as previously described^26^ with a final serum concentration of 75%. Responses were graded from 1 to 6. Strains were categorised as sensitive [3-hours count <10% of the inoculum, i.e. grade 1 (1-hour count <10%) and grade 2 (1-hour count between 10% and 100%)], intermediate [1-hour count between 10% and 100%, i.e. grade 3 (3-hours count between 10% and 100%) and grade 4 (3-hours count >100%)] or resistant [1-hour count >100%, i.e. grade 5 (3-hours count between 10% and 100%) and grade 6 (3-hours count >100%)]. Isolates were tested three times.

### Proteolytic activity assay

Proteolytic activity of the culture supernatants was assessed as previously described^27^. Skim milk agar plates containing 10% of skim milk and 1% of agar were prepared. Supernatants of bacterial cultures were added into 9 mm diameter punched holes in skim milk agar and incubated at 37 °C for 24 h. Proteolytic activity was measured by the diameter of the clear zone surrounding the holes. Three independent experiments were performed for each isolate.

### Pyocyanin quantification assay

After overnight incubation at 37 °C, 3 mL of chloroform were added to 5 mL culture supernatant and mixed vigorously. One milliliter of 0.2 M hydrochloric acid was added to the organic layer and the absorbance was measured at 520 nm. Pyocyanin concentration (mg/L) was calculated as: *P* = (*OD* × *17*.*072*) *x* (*5/3*) where *OD* is optical density at 520 nm, *17*.*072* is the pyocyanin extinction coefficient, and *5/3* is the dilution factor^28^. Three independent experiments were performed for each isolate.

### Cytotoxicity assay

A549 human lung cells monolayers seeded in 24-well tissue culture plates were infected by bacterial suspensions (multiplicity of infection of 100). After 6 hours of incubation, cytotoxicity towards A549 cells was quantified by measuring the amount of lactate dehydrogenase (LDH) released into the culture supernatant using the CytoTox 96^®^ Non-Radioactive Cytotoxicity Assay kit (Promega^®^). Cytotoxicity was calculated relative to mortality of uninfected cells (set at 0%) and mortality of cells lysed with Triton X-100 1% (positive control, 100%). Assays were carried out in triplicate in three independent experiments for each isolate.

### Twitching motility assay

Twitching motility was studied as previously described^29^. Single colonies were inoculated to the bottom of a 1% LB agar plate. The plates were incubated for 24 hours at 37 °C. The agar was carefully removed after incubation and the adherent bacteria stained with crystal violet dye, followed by washing with tap water to remove unbound dye. Twitching zone areas were measured. Three independent experiments were performed for each isolate.

### Biofilm assay

For each isolate, a 0,5 Mac Farland suspension was prepared in BHI, diluted 1/100, and used to inoculate four wells of a 96-well polystyrene microtiter plate. After 24 H at 30 °C without shaking, the bacterial suspensions were removed, the wells were washed with physiological water, air-dried for 20 mn and stained with Gram’s safranin solution for 5 mn. The plates were washed four times to wash off unbound safranin and air-dried for one hour. The adherent bacteria (biofilm) were then dissolved in 200 microliters of ethanol, and the absorbance at 560 nm was measured. The biofilm assay was performed three times independently.

### Statistical analysis

When appropriate, results were expressed as mean ± standard deviation. Comparative analyses were performed with the Kruskal–Wallis test. Data were analysed using GraphPad Prism v6.0 (Graphpad Software, San Diego, CA, USA). All analyses were two-tailed. A *p*-value < 0.05 was considered statistically significant.

## Results

### Genomic characterisation of *Pseudomonas aeruginosa* isolates

Three isolates per patient for a total of 4 patients were sequenced and genomes characteristics investigated. Phylogenetic analysis and MLST typing showed that in each patient individually, the 3 *P*. *aeruginosa* isolates were very closely related. However, the isolates belonged to different clades from one patient to another (Supplementary Fig. S1).

**Figure 1:**
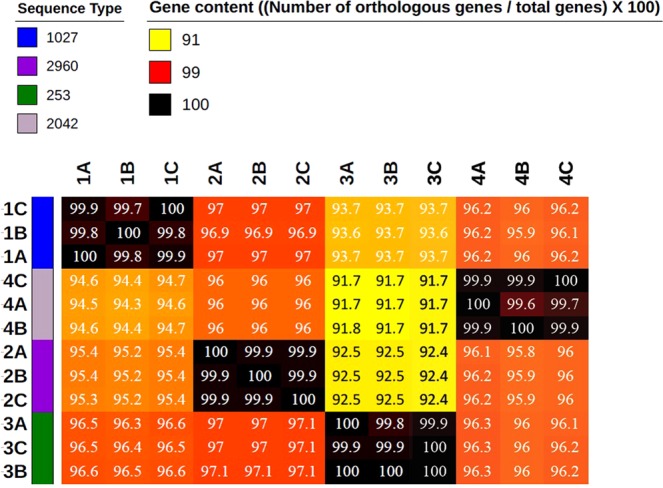
Pan genomic analyses of the 12 isolates: heatmap representation of the gene content comparison between all isolates. The gene content is calculated as the number of orthologous genes between two isolates/the total number of genes.

To specify the relationship between those isolates, we performed pan genomic analysis of the 12 genomes. Isolates from the same patient shared more than 99% of their gene content. However, strains from different patients shared between 91.7% (PA-VAP-3 *versus* PA-VAP-4) and 97.1% (PA-VAP-2 *versus* PA-VAP-3) (Fig. 1). We predicted a total of 3,854 (79.45%) orthologous genes representing the core genome (genes shared by all isolates) and 1034 orthologous genes and singletons representing the accessory genome (genes encoded in one or more isolates but not in all). *P*. *aeruginosa* accessory genome encoded mostly proteins involved in an unknown function (38.72%), products involved in DNA and RNA processing (12.90%), cell wall biogenesis (12.51%) and metabolism and transport (34.94%). Comparative analysis of the 12 *P*. *aeruginosa* genomes revealed that strains from each patient harboured several strain-specific genomic regions. Isolates from patient 1, 2, 3 and 4 encoded respectively 169, 118, 337 and 187 unique genes (only present in these genomes). Most of these genes had unknown functions (93.4%, 94.1%, 93.1% and 92.5%, respectively).

We further analysed SNPs and short indels between the earliest isolate and late isolates from each patient by mapping the reads of late isolates to the genome of earliest isolate. All the detected variations were non-synonymous SNPs, deletions or insertions (Table 1). Some of these variations affected genes involved in antibiotic resistance (*ampD*, *oprD*, *mexR*). Others concerned genes involved in bacterial virulence and/or fitness (*lasR*, *gacA*, *groEL*, *pilM*, *pilE*, *algB*, *rhlR*, *rhlB*, *rhlA*, *mpl*).

### Antimicrobial susceptibility

Prediction of resistance genes using ResFinder 3.0 showed that all 12 isolates harboured genes involved in resistance to beta-lactams (*bla*_OXA-50_ and *bla*_PAO_), aminoglycosides (*aph*(*3*′)*-IIb*), phenicols (*catB7*) and fosfomycin (*fosA*). Besides, for each patient, one of the sequential isolates showed a different antibiotic susceptibility pattern. For most patients, the emergence of resistance was correlated with the antibiotics dispensed (Table 1). Thus, patient 1 was treated with ceftazidime after isolate 1A was recovered. Isolate 1C exhibited increased resistance to beta-lactams, explained by a nonsense mutation in *ampD*. Moreover, strains collected from patients 2 and 3 acquired resistance to imipenem, explained by insertion (isolate 2B) or deletion (isolate 3C) in *oprD*. Whereas a carbapenem treatment was administered to patient 3, surprisingly, patient 2 did not receive imipenem. Finally, patient 4 was treated with cefepime and levofloxacin after isolate 4A was collected. Isolate 4B showed increased resistance to beta-lactams and fluoroquinolones, and harboured an insertion in *mexR*.

### Resistance to human serum

Strains from the 4 patients displayed very different levels of serum resistance (Table 2). In patient 2, all isolates escaped to serum killing, with a 3-hours count >100% of the inoculum whereas in patients 1 and 3, all isolates were very sensitive to serum (1-hour and 3-hours count <10%). In addition, a significant difference was detected between isolates from patient 4. The isolate 4B, a *lasR* mutant, was more sensitive to serum bactericidal action than isolates 4A and 4C (1-hour and 3-hours counts <100% for isolate 4B *vs* all counts >100% for isolates 4A and 4C).

**Table 2:** Resistance to human serum, proteolytic activity, pyocyanin production, cytotoxicity, twitching motility and biofilm production of the 12 isolates. Arrows indicate *lasR* mutants. Stars indicate *exoU* positive isolates.

Isolate	Grades of response in serum bactericidal assays^*a*^	Proteolytic activity: clear zone diameter (mm)	Pyocyanin quantification (mg/L)	Cytotoxicity on A549 cells (%)	Twitching: motility area^*b*^	Biofilm production: OD 560 nm
***Patient 1*** **(** ***Strain PA-VAP-1*** **)**						
1A*←	1	9.0 ± 0.0	0.00 ± 0.00	104.00 ± 4.00	0	0.13 ± 0.06
1B*←	1	9.0 ± 0.0	0.02 ± 0.02	100.20 ± 9.19	0	0.40 ± 0.36
1C*←	1	9.0 ± 0.0	0.15 ± 0.05	104.20 ± 10.48	0	1.85 ± 0.37
***Patient 2*** **(** ***Strain PA-VAP-2*** **)**						
2A	4	12.7 ± 0.6	3.28 ± 1.64	26.53 ± 10.27	+	0.45 ± 0.12
2B	4	12.5 ± 0.5	3.05 ± 1.47	62.78 ± 31.31	+	0.63 ± 0.17
2C	4	14.3 ± 0.3	3.35 ± 1.22	75.25 ± 43.76	++	0.55 ± 0.11
***Patient 3*** **(** ***Strain PA-VAP-3*** **)**						
3A*	1	15.2 ± 1.0	6.73 ± 0.67	107.70 ± 8.45	++	1.57 ± 0.36
3B*←	1	9.0 ± 0.0	0.38 ± 0.66	109.47 ± 13.99	0	0.62 ± 0.35
3C*←	1	9.0 ± 0.0	0.31 ± 0.44	104.90 ± 11.30	0	0.53 ± 0.35
***Patient 4*** **(** ***Strain PA-VAP-4*** **)**						
4A	6	14.2 ± 1.3	3.18 ± 0.56	82.3 ± 17.91	++	0.90 ± 0.16
4B←	3	9.0 ± 0.0	0.02 ± 0.04	6.86 ± 5.72	0	0.77 ± 0.14
4C	6	13.0 ± 0.0	3.74 ± 0.72	81.03 ± 6.52	++	0.64 ± 0.35

^*a*^Sensitive = grades 1 and 2; Intermediate = grades 3 and 4; Resistant = grades 5 and 6.

^*b*^0, no twitching motility; +, 1–10 mm; ++, 10–20 mm.

### Proteolytic activity assay

Important differences were observed between the isolates for 2 patients (Table 2). Proteolytic activity of isolate 3A was higher than isolates 3B and 3C, devoid of protease activity and *lasR* mutants. Similarly, proteolytic activity of isolates 4A and 4C was higher than isolate 4B, a *lasR* and *rhl* mutant which did not show any protease activity. Isolates collected in patient 1 did not show any proteolytic activity, in correlation with a 113-bp deletion in *lasR* for the 3 isolates (compared to reference strain PAO1).

### Pyocyanin quantification assay

Important differences were detected between the isolates for 2 patients (Table 2). Pyocyanin production by isolate 3A was higher than *lasR* mutants isolates 3B and 3C (although the difference was not statistically significant with *p* = 0.06). Similarly, isolates 4A and 4C produced more pyocyanin than *lasR* and *rhl* mutant isolate 4B (although *p* = 0.06).

### Cytotoxicity assay

Differences in cytotoxic activity were observed, both interpatient and intrapatient (Table 2). *ExoU* positive isolates had higher levels of cytotoxicity than others (*p* < 0.01). Besides, a difference appeared between the *exoU* negative isolates from patient 4. Isolate 4B, *lasR* and *rhl* mutant, was less cytotoxic than isolates 4A and 4C (although the difference was not statistically significant with *p* = 0.07).

### Twitching motility assay

No twitching motility was detected for all *lasR* mutants (Table 2). Based on the 3 experiments performed, isolates 2A and 2B were less mobile than other isolates with intact *quorum sensing*. However, the isolate 2C, which harboured a nonsense mutation in *pilM* and an insertion in *pilE*, recovered twitching motility (with a motility area 3 times larger compared with isolates 2A and 2B).

### Biofilm assay

Important differences were detected between the isolates for 2 patients (Table 2). Biofilm production was higher for isolate 3A than for isolates 3B and 3C (*p* = 0.02), harbouring an insertion in *algB*. Furthermore, isolate 1C, which exhibited a non-synonymous mutation in *gacA*, produced more biofilm than isolates 1A and 1B (*p* = 0.02).

## Discussion

In this work, we studied *P*. *aeruginosa* isolates collected sequentially in 4 patients with VAP over periods of 12 days to 5 weeks. As shown by whole genome sequencing, each patient was infected with one strain, distinct from strains found in other patients. We highlighted an intrahost diversification of these 4 strains over a short period.

On the one hand, *P*. *aeruginosa* developed antibiotic resistance in all patients, correlated with the antibiotics dispensed. The acquisition of resistance mechanisms matched genetic modifications detected by whole genome sequencing. Thus, isolate 1C exhibited increased resistance to beta-lactams, explained by a nonsense mutation in *ampD*. Indeed, *ampD* inactivation is known to lead to hyperproduction of beta-lactamase AmpC^30^. Moreover, strains collected from patients 2 and 3 acquired resistance to imipenem, explained by genetic variations in *oprD*. Actually, the main mechanism of imipenem resistance in *P*. *aeruginosa* is alteration or decreased production of outer membrane porin OprD^31^. Finally, isolate 4B showed increased resistance to beta-lactams and fluoroquinolones, and harboured an insertion in *mexR*. It has been shown that premature termination of MexR leads to overexpression of MexAB-OprM efflux pump^32^, of which beta-lactams and fluoroquinolones are substrates. In a study by Wang *et al*., an epidemic ST1971 *P*. *aeruginosa* strain underwent rapid evolution during VAP infections. They described emergence of *mpl* mutants under beta-lactam selective pressure in 3 patients. Some of these mutants showed increased resistance to ceftazidime^13^. In our study, we observed the appearance of a *mpl* mutant in patient 3 under beta-lactam treatment. However, the V105G substitution in Mpl protein (UDP-N-acetylmuramate:L-alanyl-γ-D-glutamyl-meso-diaminopimelate ligase) was not related to increased resistance to ceftazidime.

On the other hand, we detected genetic events associated with changes in bacterial virulence and especially emergence of *quorum sensing* deficiency. Indeed, *quorum sensing* mutants appeared in patients 3 and 4. Isolates 3B and 3C were *lasR* mutants, while isolate 4B was a *lasR* and *rhl* system double mutant (Table 1). Besides, all 3 isolates from patient 1 were *lasR* mutants. These 6 isolates displayed reduced production of *quorum sensing*-dependent virulence factors (pyocyanin and protease), but also decreased serum resistance, cytotoxicity on A549 cells (except for *exoU* positive isolates) and twitching motility (Table 2). It is noteworthy that isolate 4C, collected in a blood culture 2 days after isolate 4B, was not *quorum sensing* deficient and therefore retained serum resistance.

*Quorum sensing* mutants have been poorly described apart from chronic *P*. *aeruginosa* infections. Three studies have reported such mutants in lung colonisation of mechanically ventilated patients, in highly variable proportions (19 to 76% of *P*. *aeruginosa* strains)^33–35^. *Quorum sensing* mutants were frequently recovered in association with wild-type isolates and their proportion increased in patients over time in one study^34^. Furthermore, two studies identified *quorum sensing* deficient isolates in *P*. *aeruginosa* acute pneumonia, but in less than one third of patients^36,37^. As in our work, mutants evolved in parallel towards increased antibiotic resistance in the study by Karatuna *et al*^37^. Besides, Wang *et al*. described quick evolution towards *quorum sensing* deficiency during VAP, as in our study^13^. They showed that a deletion causing a LasR deficient phenotype slightly impaired the *in vivo* virulence of *P*. *aeruginosa* in a murine pulmonary infection model. Considering the divergent data found from literature, the virulence of the *quorum sensing* mutants may actually vary depending on the type of infection. In a study by Köhler *et al*., VAP occurred less frequently in patients colonised with *quorum sensing* mutants than in patients colonised with non-deficient *P*. *aeruginosa*^35^. On the opposite, among 100 *P*. *aeruginosa* isolates responsible for corneal ulcers, 22% were *lasR* mutants and were associated with worse patient outcomes^38^. In our study, among the 3 patients infected with *quorum sensing* mutants, one died in intensive care unit and 2 could discharge, while the patient without *quorum sensing* mutant died in intensive care unit. Given the small number of patients, it is here impossible to conclude about the intrahost virulence of *lasR* mutants. As a matter of fact, it is difficult to define their pathogenicity *in vivo* since they are probably present in combination with the original non-mutated isolates. Thus, “cheaters” have a fitness advantage as they benefit from *quorum sensing*-dependent molecules without paying the metabolic cost. The true prevalence of these mutants and their virulence during acute infections in critically ill patients should be further explored, taking into account a potential heterogeneity of the *P*. *aeruginosa* population in clinical samples.

In the study by Wang *et al*., convergent evolution towards attenuated virulence of *P*. *aeruginosa* was largely explained by pyoverdine deficiency in 3 VAP patients. They detected mutations in *pvdS*, which encodes a sigma factor that coordinates the expression of multiple proteins involved in pyoverdine synthesis and many virulence factors^13^. Pyoverdine-deficient mutants were isolated after 24 to 78 days of mechanical ventilation, at later stages than *quorum sensing* mutants. We did not find evolution towards *pvdS* modifications despite an observation period of more than 30 days in some patients.

However, we discovered emergence of a non-synonymous SNP in *gacA* for patient 1 strain (Table 1) as in the study by Wang *et al*. GacA is the response regulator of the GacA/GacS two-component system that regulates expression of a large number of genes and especially promotes biofilm formation^39^. The S201A substitution detected in GacA for isolate 1C was associated with increased biofilm formation (Table 2). Conversely, the strain infecting patient 3 evolved to reduced biofilm production, which was correlated with a 10-bp insertion in *algB*. Indeed, transcriptional activator AlgB regulates alginate production in *P*. *aeruginosa*^40^. Thus, in our study, biofilm production did not seem to play a relevant role in recurrence of VAP, keeping in mind that its regulation depends on a large number of factors and that its *in vitro* assessment is quite difficult.

Other variations were detected in genes known to have a crucial role in *P*. *aeruginosa* virulence. A non-synonymous SNP in *groEL* arose in strain from patient 2 (Table 1). GroEL is a chaperonin protein, homolog of heat shock protein 60. Shin *et al*. demonstrated that secretion of GroEL by *P*. *aeruginosa* stimulated the release of pentraxin PTX3 by human monocytes, promoting innate immune response^41^.

Lastly, isolate 2C harboured a nonsense mutation in *pilM* and an insertion in *pilE*, encoding subunits of type IV pili. This *P*. *aeruginosa* major virulence factor contributes to adhesion, biofilm formation, extracellular protein secretion and flagellum-independent twitching motility^42^. We found that variations in *pilM* and *pilE* were correlated with enhanced twitching motility (Table 2). It could be assumed that these modifications in the type IV pili structure improved its flexibility.

In conclusion, we clearly proved that pathoadaptation occurred over a short period (from 10 to 25 days) in 4 different non-hypermutable *P*. *aeruginosa* strains. These intrahost genetic and phenotypic modifications under antibiotic selection pressure in the context of VAP combined antimicrobial resistance development and changes in bacterial virulence. We particularly highlighted a convergent trend towards *quorum sensing* deficiency as in the recent study of Wang *et al*.^13^. Further studies are required to investigate the prevalence of *quorum sensing* mutants in various types of acute infection as well as their link with antibiotic treatment and their pathogenicity.

## Supplementary information

Supplementary Figure S1

## Data Availability

The whole-genome shotgun sequencing data for *P*. *aeruginosa* PA-VAP-1A to PA-VAP-4C have been deposited at Genbank under accession numbers CP028332, CP028331, CP028330 and CP028368.
