# Supplementary material for: Rapid genetic and phenotypic changes in Pseudomonas aeruginosa clinical strains during ventilator-associated pneumonia
Source: Sci Rep. 2019 Mar 18;9:4720. doi: 10.1038/s41598-019-41201-5 (PMC6423012; doi:10.1038/s41598-019-41201-5)
Supplement: Supplementary file 1 — Supplementary Figure S1 [file 41598_2019_41201_MOESM1_ESM.pdf]

Rapid genetic and phenotypic changes in *Pseudomonas aeruginosa* clinical strains during ventilator-associated pneumonia

Elise Persyn[1,2], Mohamed Sassi[3], Marc Aubry[4], Martin Broly[2], Sandie Delanou[1], Karim Asehnoune[1,2], Nathalie Caroff[1], Lise Crémet[1,2]

[1]EA3826 Université de Nantes, IRS2 Nantes Biotech

[2] CHU Nantes, 9 quai Moncousu, Nantes Cedex 1, F-44093 France

[3]Inserm U835, Université de Rennes

[4]Université de Rennes, CNRS, IGDR [(Institut de génétique et développement de Rennes)] - UMR 6290, F-35000 Rennes, France ; Université de Rennes, Plateforme GEH, CNRS, Inserm, BIOSIT - UMS 3480, US_S 018, F-35000 Rennes, France

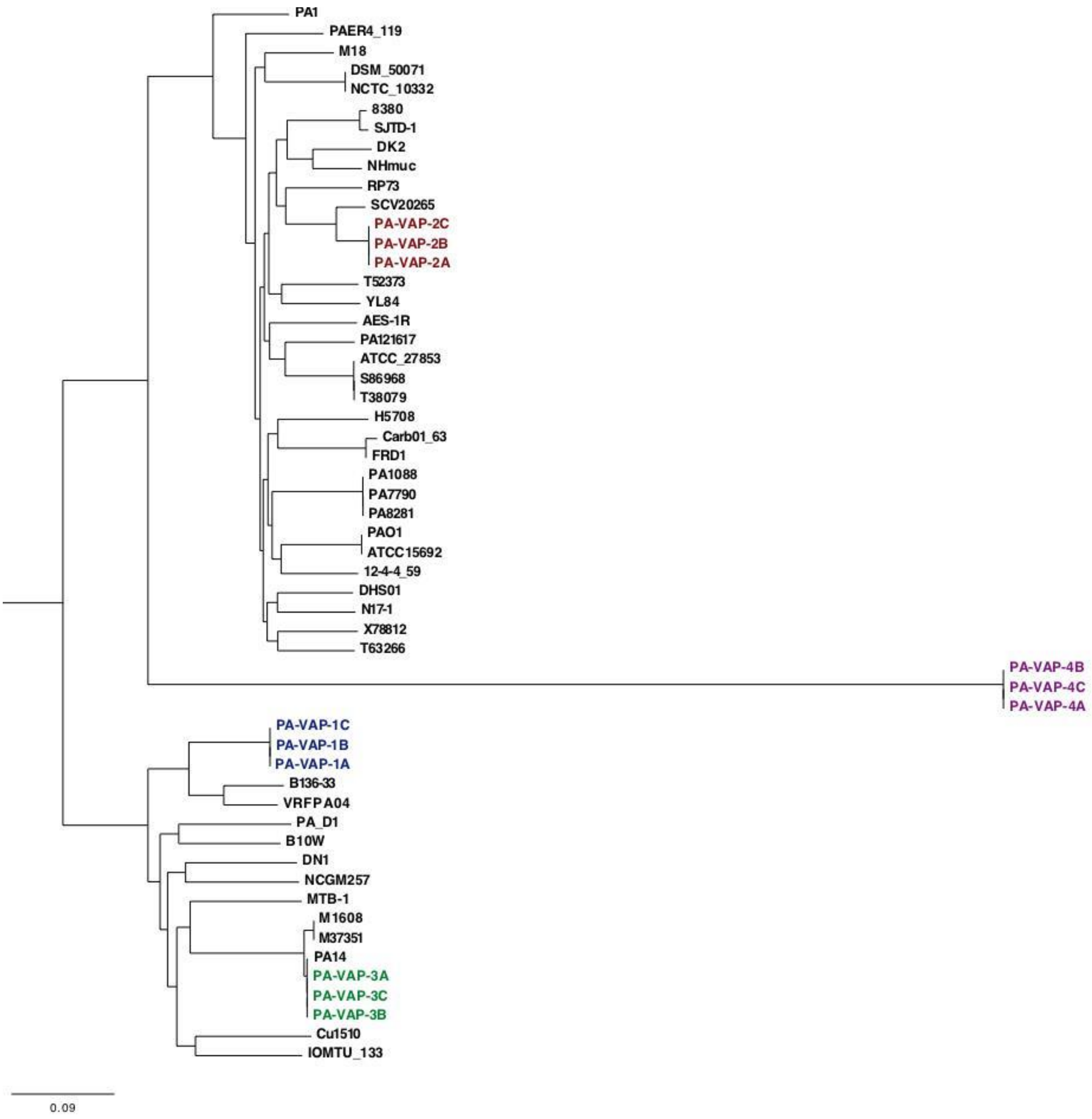

Supplementary Figure S1. Phylogenetic analysis of the 12 isolates and 43 *P. aeruginosa* genomes
